# Primary clear cell sarcoma of the femur: a unique case with RT-PCR and direct sequencing confirmation of *EWSR1/ATF1* fusion gene

**DOI:** 10.1186/s12891-021-03969-4

**Published:** 2021-01-21

**Authors:** Yuta Kubota, Kazuhiro Tanaka, Masanori Hisaoka, Tsutomu Daa, Tatsuya Iwasaki, Masanori Kawano, Ichiro Itonaga, Hiroshi Tsumura

**Affiliations:** 1 Department of Orthopaedic Surgery, Faculty of Medicine, Oita University, 1-1 Idaigaoka Hasama, 879-5593 Yufu City, Oita Japan; 2 Department of Pathology and Oncology, School of Medicine, University of Occupational and Environmental Health, 1-1 Iseigaoka, Yahatanishi‐ku, 807-8555 Kitakyushu, Japan; 3 Department of Diagnostic Pathology, Faculty of Medicine, Oita University, 1-1 Idaigaoka Hasama, 879-5593 Yufu City, Oita Japan

**Keywords:** Clear cell sarcoma, Primary bone tumor, Melanoma, Fusion gene, Direct sequencing, Reverse transcription polymerase chain reaction

## Abstract

**Background:**

It is very rare for clear cell sarcomas (CCS) to arise in the bone. During diagnosis, it is important to distinguish primary CCS of bone from bone metastasis of melanoma because this difference fundamentally changes the therapeutic options. Recently, characteristic fusion genes of CCS have been detected using reverse transcription polymerase chain reaction (RT-PCR) or direct sequencing which allowed to distinguish CCS from melanoma. However, there was no study applying these analyses with positive results. In this case, we describe the use of fusion gene analysis to diagnose a primary CCS of the bone.

**Case presentation:**

A 36-year-old male presented with a four-months history of left knee pain. Magnetic resonance imaging showed a lesion in the left femoral medial epicondyle. Histological examination of the biopsy specimen revealed proliferating oval or rounded cells. These cells had clear cytoplasm arranged in fascicles or compact nests with frequent deposits of brown pigment. Furthermore, immunohistochemistry analysis revealed that tumor cells were positive for S-100 protein, HMB-45, Melan-A, and SOX10. It stained negative for CD34 and BRAF v600e. Conclusively, detection of the *EWSR1/ATF1* fusion gene using RT-PCR and direct sequencing confirmed that the lesion was a primary CCS of the bone. Wide-margin resection and reconstruction with a tumor endoprosthesis were performed.

**Conclusions:**

Herein, we diagnosed a rare case of primary CCS of the bone by detecting *EWSR1/ATF1* fusion gene using RT-PCR and direct sequencing. Since fluorescence-in situ hybridization (FISH) and RT-PCR could show false positive by mainly due to technical problems, it is better to perform direct sequencing to confidently diagnose the tumor as a primary CCS especially at very rare site such as bone.

## Background

Clear cell sarcoma (CCS) was first described by Enzinger in 1965 [1]. It is a malignant soft tissue tumor arising from tendons and aponeuroses. CCS has limited treatment options because effective radiotherapy and chemotherapy regimens have not been established for this type of tumor. The five-year survival rate of CCS is 47 % and the 10-year survival rate is only 36 %, [2] demonstrating the aggressive nature of this tumor. CCS is rare, and accounts for less than 1 % of soft-tissue sarcomas [1]. It is very rare for CCS to be localized in the bone. To our knowledge, there are currently only 13 reports in English describing primary CCS of the bone (Table 1) [3–15]. The first primary CCS of the bone was reported in the right ulna by Yokoyama et al. [3]. They diagnosed the neoplasm as CCS on the basis of both histopathological and immunohistochemical features including the presence of S-100 protein, HMB-45, and vimentin. Their findings were consistent with those of both melanoma and CCS, but they seemed to be more closely related to CCS. However, their examination findings were as a result of techniques that did not involve cytogenetic analysis, which was not commonly used and the appropriate method for which had not been established until then; therefore, they could not definitely rule out melanoma as the diagnosis [3, 16]. Panagopoulos et al. [17]. examined *EWS/ATF1* fusion genes in CCS of soft tissue using reverse transcription polymerase chain reaction (RT-PCR) amplification and sequence analysis in 2002. Coindre et al. [18]. detected *EWS/ATF1* fusion transcripts in 38 paraffin-embedded CCS tissues out of 41 interpretable samples (93 %) in 2006. This study showed that RT-PCR on paraffin-embedded tissues was useful for distinguishing CCS from melanoma. Furthermore, RT-PCR demonstrated that *EWSR1/CREB1* fusion gene was another fusion gene of CCS [19]. The *EWS/ATF1* fusion gene has also been detected using fluorescence in-situ hybridization (FISH) in bone CCS samples [10, 11, 15]. In contrast, there are no reports confirming whether the *EWS/ATF1* or *EWSR1/CREB1* fusion genes can be detected using direct sequencing. In this article, we report a case of CCS in the femur with the *EWS/ATF1* fusion gene detected using direct sequencing and RT-PCR.

**Table 1:** Reported cases of primary CCS of bone (n = 13)

Author	Age/Sex	Location	General screening for primary lesion including melanoma	Immunohistochemistry	Genetic analysis	Treatment	Follow up
Yokoyama et al. [3]	33/F	Right ulna	Various radiograph	Positive: S-100, HMB-45, vimentin Negative: desmin, keratin	Not performed	Neoadjuvant: Ifosfamide, cisplatin and doxorubicin Wide margin resection Adjuvant: cisplatin and doxorubicin	CDF, 65 months after surgery
Brekke et al. [4]	62/F	Right first metatarsal	^99m^Tc MDP bone scan	Positive: S-100 protein, vimentin Negative: HMB-45, cytokeratin (AE1/AE2)	Not described	Syme’s amputation	CDF, 15 months after surgery
Gelczer et al. [5]	18/M	Left ninth rib	CT, ^99m^Tc MDP bone scan, bone marrow aspirate	Positive: S-100 protein, HMB-45	Not described	Neoadjuvant: Performed but details were not described Wide resection Adjuvant: doxorubicin, cyclophosphamide, vincristine	CDF, 55 months after treatment
Inaoka et al. [6]	55/M	Right radius	CT, skin checked by dermatologist, ophthalmofundoscopy, upper gastrointestinal endoscopy, Bone scintigraphy with ^99m^Tc-HMDP	Positive:S-100 protein, HMB-45, vimentin	Negative (method not described)	Neoadjuvant: cisplatin and doxorubicin Total tumor resection	CDF, 18 months after primary surgery
Choi et al. [7]	48/F	Right first metatarsal	CT, whole body radioisotope scan	Positive: S-100, HMB-45 Negative: cytokeratin, epithelial membrane antigen	Not performed	Below-knee amputation of the right leg	DOD, 20 months after surgery
Hersekli et al. [8]	28/F	Left ninth rib	CT, skin checked by dermatologist, Bone scintigraphy with ^99m^Tc-HMDP and ^67^Ga	Positive:S-100 protein, HMB-45	FISH: negative	Total tumor resection adjunctive radiotherapy of 500 cGy	CDF, 33 months
Kazakos et al. [9]	61/M	Left scapula	Bone scanning, CT	Positive: S-100, HMB-45, NSE, EMA, cytokeratin, myosin	Not described	Wide resection Adjuvant: ifosfamide, vincristine and epirubicin	DOD, 15 months after adjuvant chemotherapy
Rocco et al. [10]	53/M	sternum	CT, PET, bone scans	Positive: S-100, TFE3 Negative: HMB-45, MART1, cytokeratins, epithelial membrane antigen, renal cell carcinoma, CD10, chromogranins, synaptophysin, inhibin, calretinin	FISH: positive	Wide resection	Not described
Zhang et al. [11]	25/M	sacrum	CT, MRI (thorax and abdomen), Bone scintigraphy with ^99m^Tc-HMDP, skin, oral, anal and fundus oculi checked	Positive: S-100, HMB-45, Melan-A Negative: EMA, CD117, CD34, MSA, GFAP, PGM-1, RCC, MIB-1, AE1/AE3, CEA, Des, and HBME-1	FISH: positive	Curettage and debridement	AWD, 9 months after surgery
Liu et al. [12]	20/F	Proximal right humerus	Bone scintigraphy with ^99m^Tc-HMDP	Positive: S-100, HMB-45	FISH: negative	Neoadjuvant: cisplatin and doxorubicin Total tumor excision-alcoholization-replantation, internal fixation and bone cement implantation Adjuvant: cisplatin, doxorubicin and methotrexate	CDF, 1 year after treatment
Nakayama et al. [13]	81/M	Left pubic bone	CT, PET/CT, Bone scintigraphy with ^99m^Tc-HMDP, ^67^Ga-citrare scintigraphy, skin checked by dermatologist	Positive: S-100, HMB-45, Melan-A Negative: cytokeratin, epithelial membrane antigen	FISH: negative, direct sequencing BRAF mutation: negative	Dimethyl triazeno imidazole carboxamide, 1-[4-amino-2-methyl-5-pyrimidinyl]-methyl-3-[2-chloroethyl]-3-nitrosourea hydrochloride and vincristin, radiotherapy	DOD
Licata et al. [14]	42/M	Left third metatarsus	Bone scan	Positive: S-100, HMB-45, Melan-A Negative: cytokeratin, epithelial membrane antigen	Not described	transtibial amputation	Not described
Xu et al. [15]	61/M	Right calcaneus	Bone scintigraphy with ^99m^Tc-HMDP	Positive: S-100, vimentin, melanA Negative: HMB45, NSE, SMA, desmin, CD117, CD99, cytokeratin	Positive (method not described)	Below-knee amputation	CDF, at the 6 months follow-up
Kubota et al. (The present study)	36/M	Left Femur	skin checked by dermatologist, Whole-body CT, PET/CT, MRI (femur, upper limb, spine)	Positive: S-100, HMB-45, melanA, vimentin, Sox10, INI-1, EMA, CD99, TLE-1, Ki67 Negative: AE1/AE3, CD34, CD56, LCA, WT-1, BRAF v600e, H3.3G34, Fli-1, ERG, NKX2.2	RT-PCR, direct sequence of transcripts and found *EWSR1*-exon 8 forward and *ATF1*-exon 4 reverse (type 1)	Wide margin resection and distal femoral replacement using cemented rotating hinge prosthesis	CDF, 9 months after surgery

*CT *Computed tomography, *PET * Positron emission tomography, *MRI *Magnetic resonance imaging, *F *Female, *M *Male, ^99m^*Tc MDP *Tc-99m-methylene diphosphonate, *CDF *Continuous disease free, *DOD *Died of disease, *AWD *Alive with disease, *RT-PCR *Reverse transcription polymerase chain reaction, *FISH *Fluorescent in-situ hybridization

## Case presentation

**Fig. 1:**
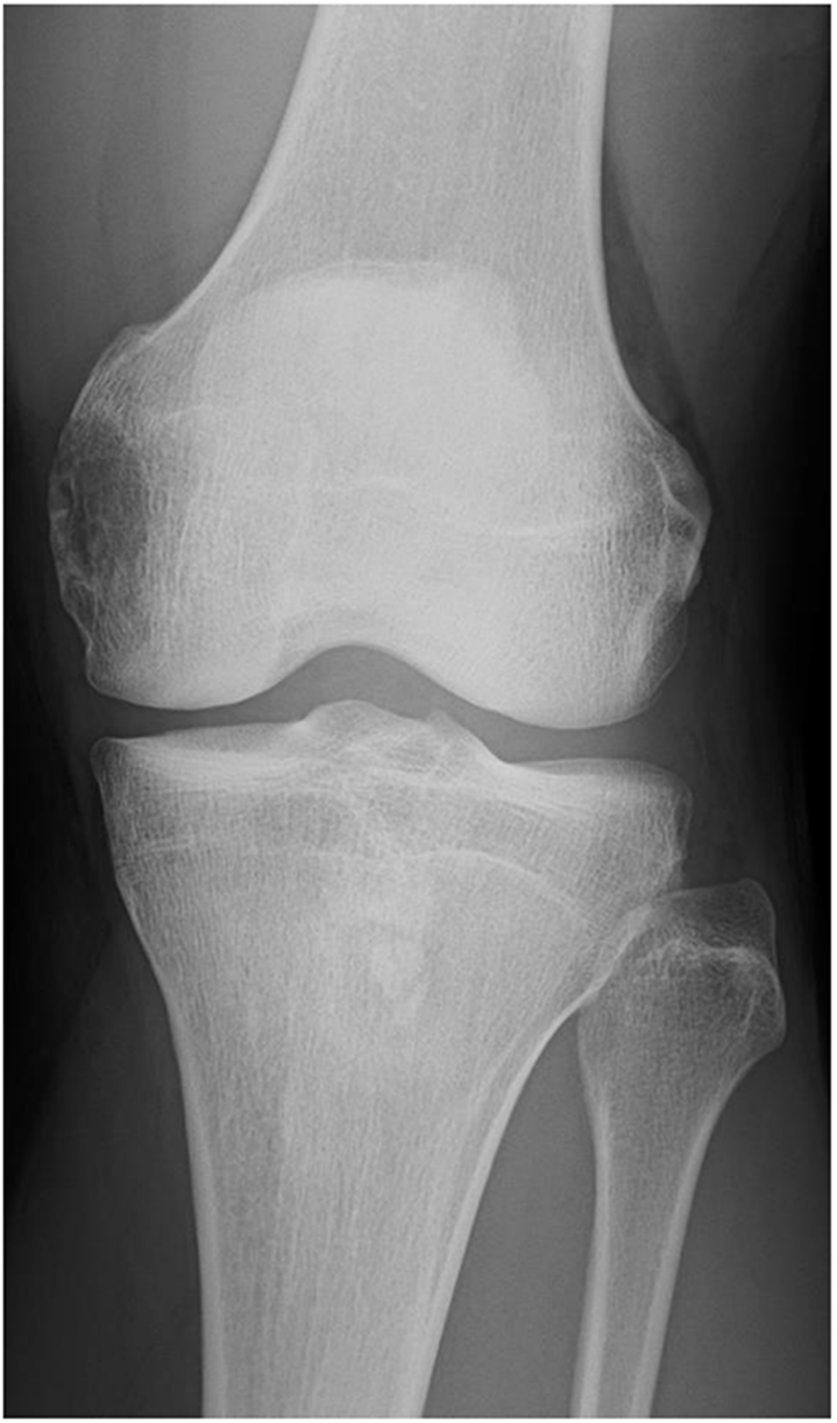
Radiograph shows osteolytic lesion in the left distal femur

**Fig. 2:**
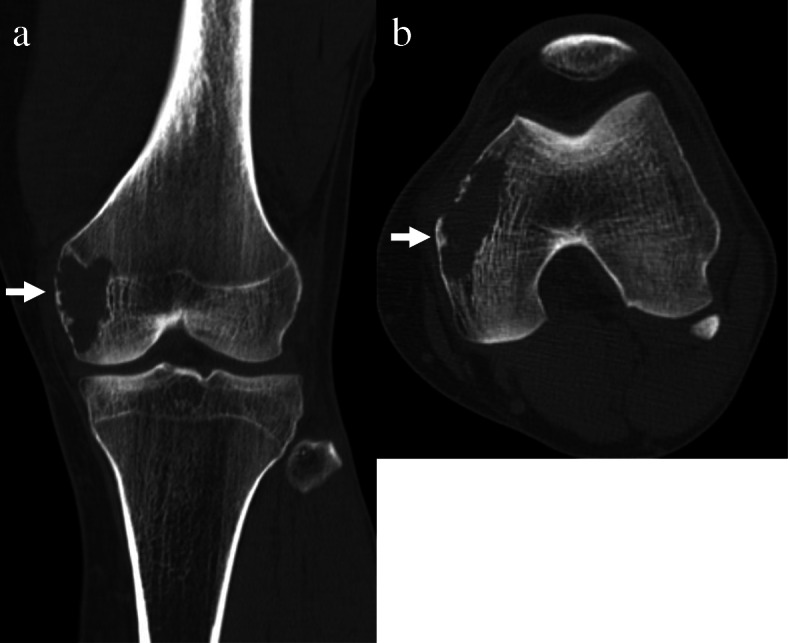
Computed tomography scans show a lytic lesion with partial cortical destruction in the distal femur. Lytic lesions (arrows) with partial cortical destruction are shown in (**a**) 3D coronal view and (**b**) Axial view

**Fig. 3:**
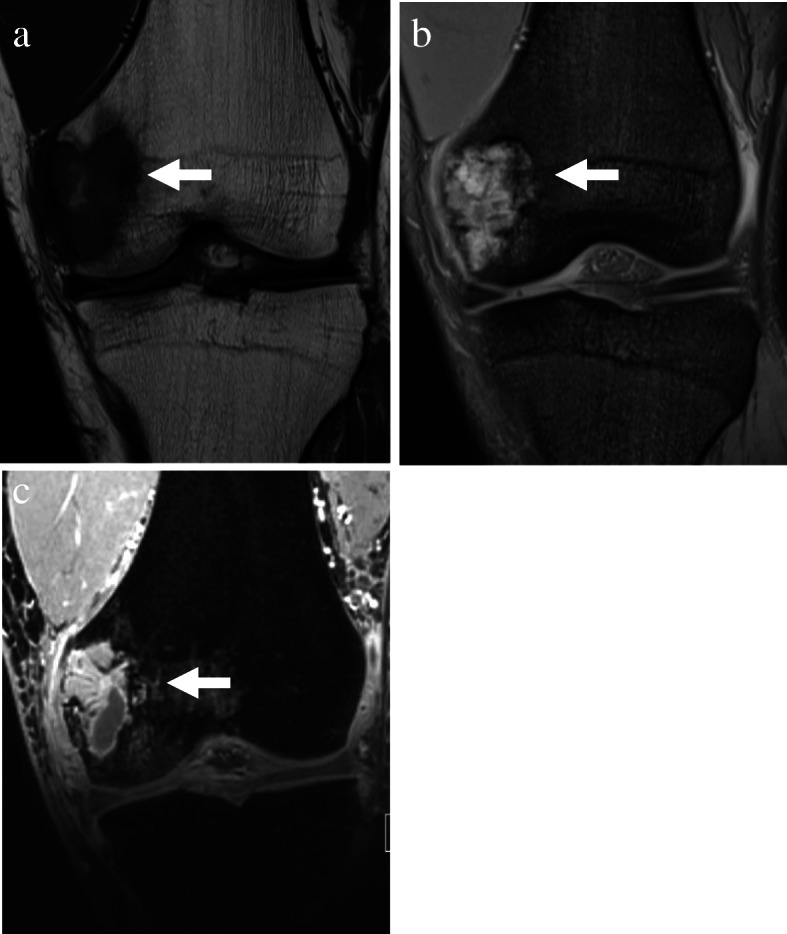
Magnetic resonance imaging shows an ill-defined mass lesion in the left distal femur. Ill-defined mass lesion appears (**a**) hypointense on T1-weighted image and (**b**) hyperintense and isointense on T2-weighted image. **c** T1-weighted image with gadolinium shows primary enhancement at mass lesion excluding small non-enhancement area

A 36-year-old male presented with a four-months history of pain in the left knee. His medical history was negative for injury, among others, as the underlying cause. Physical examination revealed a tenderness at the left femoral medial epicondyle but no swelling, redness or heat around the joint. Furthermore, there was no instability or joint contracture. Radiological examination showed an osteolytic lesion in the femoral medial epicondyle with a partially destructed cortex (Fig. 1). There was no sclerotic rim or periosteal reaction. Computed tomography (CT) revealed a 38 × 19 × 17 mm osteolytic lesion that partially destroyed and thinned the cortex (Fig. 2). There was no calcification in the mass. Magnetic resonance imaging (MRI) showed that the lesion had the most hypointense area, including both hyperintense and isointense areas heterogeneously on T1-weighted images and had hyperintense areas with septal walls on T2-weighted images. Hyperintense signal areas were observed at the femoral articular surface without extraosseous soft tissue signal change (Fig. 3). Considering all images, giant cell tumor (GCT), osteosarcoma or chondrosarcoma was suspected.

**Fig. 4:**
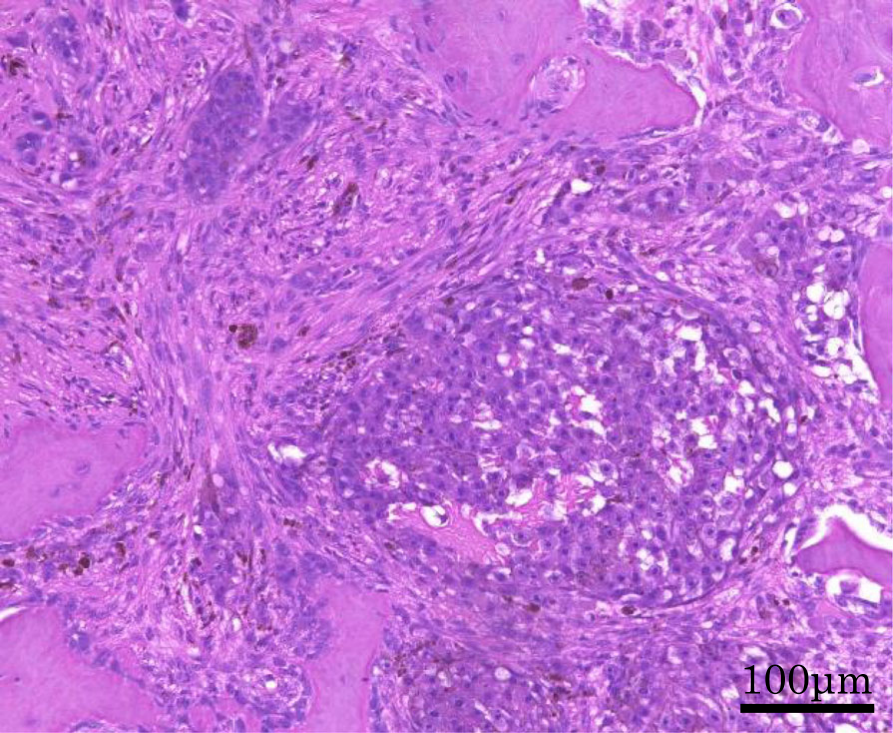
Histological examination of the biopsy specimen reveals cell proliferation in oval or rounded cells. Images show hematoxylin and eosin staining. The tumors consist of fascicles and compact nests with frequent deposits of brown pigment. Magnification: 200 ×

**Fig. 5:**
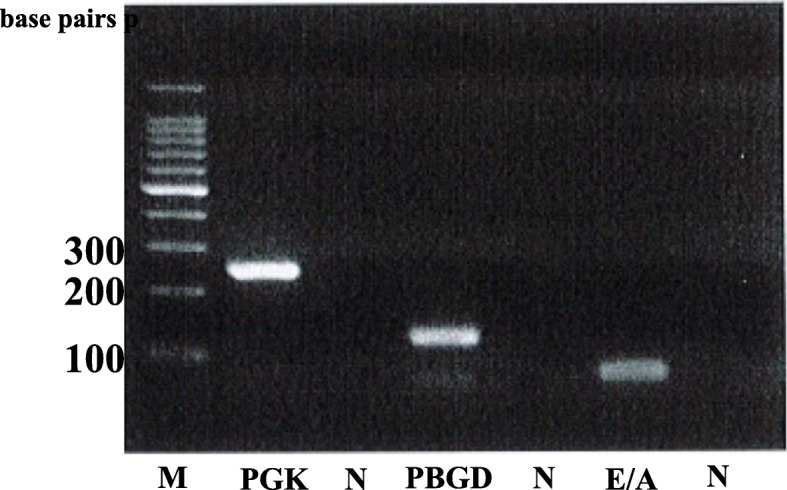
Reverse transcription-polymerase chain reaction using *EWSR1/ATF1* primer (81 base pairs). M: molecular size marker; N: negative control (distilled water); PGK: phosphoglycerate kinase, 247 base pairs; PBGD: porphobilinogen deaminase, 127 base pairs; E/A: *EWSR1/ATF1* primer, 81 base pairs

**Fig. 6:**
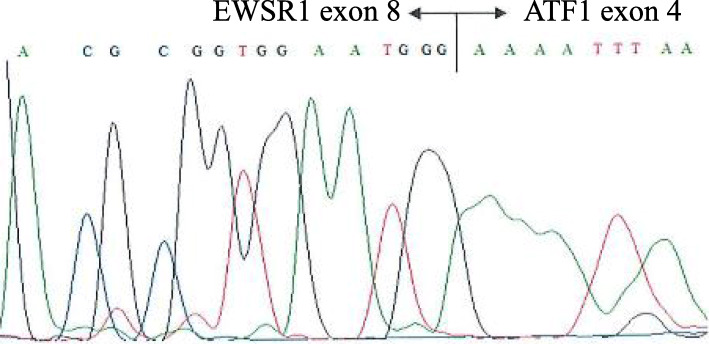
Partial sequence of the RT-PCR products corresponding to the *EWSR1/ATF1* fusion gene. RT-PCR, reverse transcription polymerase chain reaction

Open biopsy was performed for a definitive diagnosis. Histological examination revealed that oval or rounded cells were proliferating. These cells had clear cytoplasm arranged in fascicles or compact nests with frequent deposits of brown pigment (Fig. 4). For a more accurate evaluation of the tumor type, immunohistochemistry was performed using a panel of markers. This analysis revealed that tumor cells were positive for S-100 protein, HMB-45, Melan-A, and SOX10. It stained negative for CD34 and BRAF v600e. Considering these features [20], the main differential diagnoses were clear cell sarcoma and melanoma. RT-PCR and direct sequencing are the molecular techniques that help differentiate between the different *EWS/ATF1* fusion types and breakpoints [19–21]; therefore, we used both the methods. We examined the tumor for *EWSR1/ATF1* transcripts using RT-PCR (Fig. 5) and direct sequencing on the paraffin-embedded tissue (Fig. 6). The tumor was found to be positive for the *EWSR1/ATF1* fusion gene. Thus, we diagnosed the patient with primary clear cell sarcoma of the bone.

As CCS of the bone is so rare, extensive investigations were conducted to search for other metastases or primary tumors. The patient’s skin was checked by a dermatologist, but no melanoma was found. Whole-body CT and positron emission tomography (PET)/CT were performed and showed no other metastatic dissemination.

Based on our investigations, we concluded that this was a primary CCS localized to the bone. Because CCS does not usually respond to radiotherapy or chemotherapy [22],

adjuvant therapy was not applied to this patient. A wide-margin resection and reconstruction with an endoprosthesis were performed. We presumed that the tumor invaded the intra-articular area as the CT image showed a partially destroyed femoral medial epicondyle cortex. Accordingly, we performed an extra-articular knee resection including the suprapatellar bursa and joint capsule. The resected specimen had a pathologically confirmed negative margin and the tumor spread extraskeletally at the femoral medial epicondyle but not into the soft tissue around the capsule. Nine months after surgery, no local recurrence or metastases were detected.

## Discussion

The first report resembling primary malignant CCS of bone was presented by Yokoyama et al. [3] in 1996. In this first case, they did not collect material for genetic analysis and therefore, could not definitely diagnose CCS of bone without verification of the t(12;22) translocation; instead they suggested a diagnosis of either melanoma or CCS of the bone [3, 16]. It has been reported that t(12;22)(q13-14;q12) translocation was detected in 62.5 %-70 % of CCS cases, and that the tumors negative for it required histopathological diagnosis [17, 23].

It is very important to distinguish primary CCS of the bone from bone metastasis of melanoma as they share many common histopathological features; however CCS of the bone is very rare. Compared to melanoma, CCS typically lacks significant nuclear pleomorphism [24]. CCS is also usually strongly positive for HMB- 45, S-100, Melan-A, MITF, and negative for smooth muscle actin, desmin and keratin [8 19, 24, 25]. Melanoma, on the other hand, is typically positive for c-kit, CD68, S-100, HMB-45, Melan-A, throsinase, and vimentin, and negative for smooth muscle actin, desmin, chromogranin, and epithelial membrane antigen [24, 25]. However, each case of CCS varies, and the overlapping staining profiles between CCS and melanoma suggest that immunohistochemical examination alone cannot discriminate between these tumors. We used S-100 protein, HMB-45, Melan-A, SOX10, CD34, and BRAF v600e for histopathological examination. As mentioned above, S-100 protein, HMB-45, and Melan-A are usually positive in CCS and melanoma [24]. Positive staining for SOX10 is suggestive of CCS because *EWS/ATF1* activates melanocyte-specific MITF-SOX10 expression [26]. BRAF was reported to be rare in CCS [27]. but positive in clear cell melanoma and melanoma [28]. Similarly, CCS is negative for CD34 [19]. CD34 is useful to distinguish CCS from Epithelioid neoplasms with SMARCB1 and SMARCA4 deficiency or Mesenchymal tumors with *NTRK* fusions which are positive for CD34 [29].

Since 1996, diagnoses of primary CCS of the bone have been supported by further evidence including: (1) whole body screening tests such as PET-CT that showed no melanoma, [10, 13] (2) no previous history of melanoma, [7, 9] and (3) patients survived much longer than those with bone metastasis of melanoma [3, 6].

Meanwhile, *EWSR1/ATF1* and *EWSR1/CREB1* transcript fusions have been identified in CCS. [30, 31] Hisaoka et al. [19] reported that 33 CCSs analyzed using RT-PCR were positive for transcripts of either *EWSR1/ATF1* type 1,2,3,4, or *EWSR1/CREB1*. The case we have presented here was positive for the type 1 fusion transcript of *EWSR1/ATF1*, consisting of the forward *EWSR1*/exon 8 and reverse *ATF1*-exon 4.

To our knowledge, 13 cases of primary CCS of the bone have been reported in the literature, as shown in Table 1. The first genetic analysis was conducted by Rocco et al. in 2009 [10]. Although they confirmed rearrangement of the *EWS* gene localized on chromosome 22q12 using FISH, fusion transcripts were not detected. When seven of the 13 known cases of primary CCS of the bone were assessed for chromosomal translocation by cytogenetic analysis including FISH, only three cases were positive [10, 11, 15]. Of the four negative cases, the one reported by Inaoka et al. [6] was deemed a primary CCS of bone rather than melanoma as their patient survived for more than 18 months, which is significantly longer than the mean survival of 4.7 months for patients with melanoma [32]. Two other cases by Hersekli et al. [8] and Liu et al. [12] concluded on primary CCS of the bone based on morphological and immunoenzymatic features only. The last case described by Nakayama et al. [13] reported that whole body screening (CT, PET/CT), bone scintigraphy, and a skin check by a dermatologist were all negative for primary melanoma; therefore, their case was diagnosed as primary CCS of the bone. All cases describing the method of cytogenetic analysis used FISH. Nakayama et al. [13] also performed *BRAF* (exons 11 and 15) mutation analysis using direct sequencing.

However, there were possibilities that RT-PCR and FISH produce false positive results due to technical problems although it might be rare, [33–38] and that not all the best probes and primers for known fusion genes with optimal conditions were used in all 13 cases. Thus, we are still unable to definitively conclude that all thirteen reported cases were primary CCS of the bone. 

Furthermore, dual-color, break-apart FISH using break-apart rearrangement specific for EWSR1 gene on 22q13 is usually used for distinguishes clear cell sarcoma of soft tissue from melanoma, [39] but using the probe does not suggest fusion types or breakpoints of *EWS* gene rearrangement [19–21]. Moreover, CCS cases with *EWS/ATF1* fusion gene but not translocation t(12;22)(q13;q12-13) have been reported [17]. FISH test might not be appropriate for these cases. On the other hand, RT-PCR amplification carryover contamination leads to false-positive PCR reactions [40], and positive and negative controls for all fusion types should be prepared for reliable results. Nevertheless, there was no report using such controls in the diagnosis of primary CCS of the bone. Therefore, it is better to perform direct sequencing to confidently diagnose the tumor as a primary CCS especially at very rare site such as bone.

Here, we have reported the first case of primary CCS of the bone diagnosed by detection of the fusion gene using RT-PCR and direct sequencing.

In this case, we confirmed there were no other primary tumors using MRI and whole-body CT scans. According to Gonzaga et al., [41] 13 of the 489 cases of CCS of soft tissue (3 %) had bone metastasis at diagnosis. These 13 cases were classified as American Joint Committee on Cancer (AJCC) stage IV and their probability of 5-year survival was 15 %, and median overall survival was 8.9 months. Kawai et al. [2] showed that the cases which first metastasis site was bone were three out of the 52 cases of CCS (5.8 %) and the median time to metastasis was 13 months. Tumors > 5 cm had a significantly higher rate of metastases (79 %) than smaller tumors (48 %). Large CCS primary tumors consistently lead to metastases. Lucas et al. [42] reported that all 12 cases with tumors larger than 5 cm developed metastases. As metastases are usually derived from larger tumors (greater than 5 cm) and bone metastases from CCS are rare, it is unlikely that a primary CCS would have been missed. Additionally, our patient had no local recurrence or metastasis for nine months after surgery. Together this allowed us to obtain a diagnosis of primary CCS of the bone.

In conclusion, to our best knowledge, this is the first case of primary CCS of the bone definitively diagnosed by detecting the fusion gene using RT-PCR and direct sequencing, and the first primary CCS of the bone arising in the femur. Because primary CCS of bone is exceedingly rare, it is important for definitive diagnosis to perform the most sensitive and accurate tests to confirm the presence of the characteristic fusion genes in order to obtain a definitive diagnosis.

## Data Availability

The datasets used and/or analyzed during the current study are available from the corresponding author on reasonable request.

## Abbreviations

CCS: Clear cell sarcoma
CT: Computed tomography
FISH: Fluorescence in situ hybridization
MRI: Magnetic resonance imaging
PET: Positron emission tomography
RT-PCR: Reverse transcription polymerase chain reaction
CDF: Continuous disease free
DOD: Died of disease
AWD: Alive with disease
